# Supplementary material for: Comparative Transcriptome Analysis of Two Kalanchoë Species during Plantlet Formation
Source: Plants (Basel). 2022 Jun 22;11(13):1643. doi: 10.3390/plants11131643 (PMC9268976; doi:10.3390/plants11131643)

Supplementary Table S1. List of overrepresented GO terms that overlap between gene clusters and their corresponding expression trend during plantlet formation in *K. daigremontiana* (*Kd*) and *K. pinnata* (*Kp*). The number that follows the species name symbol Kd or Kp represents the cluster number derived from the heat map in Figure 2 that categorised all genes into clusters based on similarity in expression patterns across different plantlet developmental stages or time points. There are 8 distinct expression trends exhibited by these genes (see Figure 3 for graphical representation of expression trends). ↘ indicates the genes were downregulated, ↗ indicates the genes were upregulated from one stage or time point to the next. The occurrence records the frequency of gene clusters in which the GO term is overrepresented. The highlighted boxes show that the specific GO term is overrepresented in gene clusters of either *K. daigremontiana* (Kd), highlighted in grey and/or *K. pinnata* (Kp), highlighted in black.

			Expression Trends																			
			1					2		3					4		5	6		7	8	
**GO Term**	**GO ID**	**Occurrence**	Kd1	Kd2	Kd7	Kp1	Kp7	Kp3	Kp6	Kd4	Kd5	Kd8	Kp8	Kp12	Kp4	Kp9	Kd6	Kp5	Kp10	Kd3	Kp2	Kp11
Response to stimulus	GO:0050896	12																				
Cellular process	GO:0009987	9																				
Developmental process	GO:0032502	6																				
Multicellular organismal process	GO:0032501	6																				
Biological regulation	GO:0065007	5																				
Metabolic process	GO:0008152	5																				
Regulation of hormone levels	GO:0010817	3																				
Reproduction	GO:0000003	3																				
Response to wounding	GO:0009611	3																				
Signaling	GO:0023052	3																				
Gene expression	GO:0010467	2																				
Response to karrikin	GO:0080167	2																				

**Expression trend** (Based on Figure 5.3)							
Trend 1 ↗↘↘	Trend 2 ↗↘↗	Trend 3 ↘↗↗	Trend 4 ↘↗↘	Trend 5 ↗↗↗	Trend 6 ↗↗↘	Trend 7 ↘↘↘	Trend 8 ↘↘↗

Supplementary Table S2. List of unique GO terms that are overrepresented in gene clusters of one species but not the other. ***K. daigremontiana*** **(*Kd*) and** ***K. pinnata*** **(*Kp*).** The trend represents specific expression patterns of genes in different clusters (See Figure 3 for graphical representation of expression trends). ↘ indicates the genes were downregulated, ↗ indicates the genes were upregulated from one stage or time point to the next.

Kd Cluster	GO Term	GO ID	P-value	Gene count	Trend	GO Term	GO ID	P-value	Gene count	Kp cluster
2	Regulation of signaling	GO:0023051	2.04E-04	19	1	Immune system process	GO:0002376	1.13E-12	36	1
						Multi-organism process	GO:0051704	6.65E-11	83	
	Cellular protein-containing complex assembly	GO:0034622	3.03E-04	1		Response to drug	GO:0042493	8.40E-10	36	
						Regulation of multi-organism process	GO:0043900	1.54E-06	21	
	Carbohydrate metabolic process	GO:0005975	3.89E-04	44		Biological process	GO:0008150	1.98E-05	521	
						Organelle organization	GO:0006996	1.42E-04	14	
7	Protein-chromophore linkage	GO:0018298	6.89E-14	12		Collagen catabolic process	GO:0030574	4.51E-04	3	
						Multi-organism process	GO:0051704	7.96E-06	32	7
	Response to radiation	GO:0009314	2.01E-07	23		Response to oxygen levels	GO:0070482	4.13E-05	11	
						Immune system process	GO:0002376	5.00E-05	13	
					2	Defence response	GO:0006952	1.71E-04	53	3
						Intracellular transport	GO:0046907	3.01E-04	3	
						Drug metabolic process	GO:0017144	7.25E-04	26	
						Regulation of biosynthetic process	GO:0009889	3.60E-05	40	6
						Xylem development	GO:0010089	6.54E-05	5	
4	Cellularization	GO:0007349	5.48E-05	4	3	Regulation of biological process	GO:0050789	2.14E-06	85	8
	Ribosome biogenesis	GO:0042254	3.65E-04	19		Nitrogen compound metabolic process	GO:0006807	2.71E-05	30	
	Sulphur compound metabolic process	GO:0006790	8.77E-04	19		Stem cell population maintenance	GO:0019827	3.33E-04	5	
	Regulation of signaling	GO:0023051	9.05E-04	16		Flavonoid biosynthetic process	GO:0009813	2.02E-09	13	12
5	Localization	GO:0051179	9.05E-04	67		Cell wall organization or biogenesis	GO:0071554	1.87E-08	33	
	Water transport	GO:0006833	2.06E-04	5		Response to auxin	GO:0009733	1.91E-06	24	
	RNA processing	GO:0006396	2.22E-04	2		Multi-organism process	GO:0051704	4.26E-04	59	
	Response to fructose	GO:0009750	2.68E-04	4		The multicellular organismal reproductive process	GO:0048609	5.49E-04	13	

8	Response to chemical	GO:0042221	3.72E-04	45		Plant organ formation	GO:1905393	6.09E-04	10	
	Response to stress	GO:0006950	3.75E-04	52		Ribonucleoprotein complex biogenesis	GO:0022613	9.80E-04	1	
						Establishment of protein localization	GO:0045184	1.07E-03	5	
					4	Protein folding	GO:0006457	1.24E-10	13	
						Pigment biosynthetic process	GO:0046148	1.34E-05	7	4
						Response to virus	GO:0009615	1.80E-04	5	
						Wax biosynthetic process	GO:0010025	1.55E-07	7	9
6	Carbohydrate transport	GO:0008643	6.57E-05	10	5					
					6	Response to inorganic substance	GO:0010035	1.87E-07	31	
						Wax biosynthetic process	GO:0010025	1.42E-05	5	
						Positive regulation of seed germination	GO:0010030	1.71E-05	5	5
						Protein complex oligomerization	GO:0051259	2.05E-05	6	
						Ion transport	GO:0006811	2.40E-04	20	
						Proteasomal ubiquitin-independent protein catabolic process	GO:0010499	6.10E-06	5	
						Cellular component organization or biogenesis	GO:0071840	2.34E-04	43	10
						Response to inorganic substance	GO:0010035	3.26E-04	19	
3	Plastid organization	GO:0009657	2.88E-07	19	7					
	Water transport	GO:0006833	2.28E-05	6						
	Regulation of flavonoid biosynthetic process	GO:0009962	1.29E-04	5						
	Response to abiotic stimulus	GO:0009628	1.52E-04	59						
					8	Terpenoid metabolic process	GO:0006721	3.53E-05	10	
						Regulation of biological process	GO:0050789	4.56E-05	95	2
						Cellular response to endogenous stimulus	GO:0071495	1.06E-04	25	
						Response to light stimulus	GO:0009416	7.05E-06	32	
						Shoot system development	GO:0048367	1.96E-04	30	11
						Multi-organism cellular process	GO:0044764	3.35E-04	8	

Expression trend (Based on Figure 5.3)

Trend 1 ↗↘↘	Trend 2 ↗↘↗	Trend 3 ↘↗↗	Trend 4 ↘↗↘	Trend 5 ↗↗↗	Trend 6 ↗↗↘	Trend 7 ↘↘↘	Trend 8 ↘↘↗

Supplementary Table S3. List of overrepresented genes in selected GO terms that are shared between two or more gene clusters in *K. daigremontiana* and *K. pinnata*. The gene ID represents Locus TAIR object ID. Gene description is adapted from the TAIR locus description. The gene function is described exclusively based on its functional relevance to plantlet formation in *K. daigremontiana* (Kd) and *K. pinnata* (Kp).

Signaling GO:0023052					
Gene ID	Gene Symbol	Description	Cluster in Kd	Cluster in Kp	Gene Function & Reference
AT1G21326		MAP kinase 4 substrate 1 (MKS1) homolog	2	1	Pathogen defence [31].
AT1G42990	BZIP60	Basic region/leucine zipper motif 60	2	1	ER stress [32].
AT2G23460	XLG1	Extra-large G protein 1	2	1	Stress response [33], disease resistance [34].
AT2G30360	CIPK11	A member of the CBL-interacting protein kinase, SOS2-like protein	2	1	Salt stress [35], drought stress [36], ABA signaling [37], cadmium stress [38].
AT2G40180	PP2C5	MAPK phosphatase	2	1	Stress response [39], seed germination [40].
AT3G17510	CIPK1	CBL-interacting protein kinase 1	2	1	Nutrient deficiency [41], osmotic stress [42].
AT3G25070	RIN4	RPM1 interacting protein 4, a member of R protein complex	2	1	ER stress [43], pathogen defence [44], [45].
AT3G46620	RDUF1	RING domain-containing E3 ligase	2	1	Salt stress [46], drought stress [47], ABA-mediated germination [47].
AT4G34410	ERF109	Ethylene response factor 109	2	1	Wounding [48], seedling growth [49], salt stress [50].
AT5G47910	RBOHD	Respiratory burst oxidase homologue D	2	1	Oxidative stress [51].
AT5G48150	PAT1	Phytochrome A signal transduction 1	2	1	Light perception [52].
AT2G20900	DGK5	Diacylglycerol kinase 5	-	1,3	Freezing stress [53].
AT3G17980	C2	C2 domain	-	1,3	ABA sensitivity [54], salt & oxidative stress [55].
AT5G20480	EFR	EF-TU receptor	-	1,3	Pathogen defence [56].
AT2G03440	NRP1	Nodulin-related protein 1	2	1,3	Heat stress [57], ER stress [58].
AT3G12500	PR3	Pathogenesis-related protein 3, encodes basic chitinase	2	1,3	Pathogen defence [59], [60].
AT1G12110	NRT1	Nitrate transporter 1	2	3	Salt stress [61].
AT1G13260	RAV1	Related to ABI3/VP1 1	2	3	Dehydration stress [62].
AT1G15100	RHA2A	Ring-H2 finger A2A	2	3	ABA signaling & drought stress [63].
AT1G19640	JMT	Jasmonic acid carboxyl methyltransferase	2	3	Drought stress [64], cold stress [65].
AT1G25560	TEM1	Tempranillo 1	2	3	Salt tolerance [66], flowering [67], [68].
AT2G27030	CAM5	Calmodulin 5	2	3	Pathogen defence [69], heat shock [70], ABA inhibition during seed germination & seedling growth [71].
AT2G38310	PYL4	Pyrabactin resistance-like 4	2	3	ABA signaling during germination [72].
AT2G40340	DREB2C	Dehydration-responsive element-binding protein	2	3	Oxidative stress [73], heat stress [74], salt stress [75], ABA

		2C			biosynthesis during germination [76].
AT4G26150	CGA1	Cytokinin-responsive GATA factor 1	2	3	Chloroplast development [77], [78], flowering [79], [80].
AT5G02810	PRR7	Pseudo-response regulator 7	2	3	Heat stress [81].
AT5G07580	ERF106	Ethylene response factor 106	2	3	Pathogen resistance [82].
AT5G25190	ESE3	Ethylene and salt inducible 3	2	3	Salt stress during germination and seedling development [83].
AT5G36930	NLR	Disease resistance protein (TIR-NBS-LRR class) family	2	3	Disease resistance [84].
AT5G47120	BI1	Bax inhibitor 1	2	3	ER stress [85], drought stress [86].
AT5G66730	IDD1	Indeterminate domain 1	2	3	Promote germination [87].
Response to wounding GO:0009611					
Gene ID	Gene Symbol	Gene Description	Cluster in Kd	Cluster in Kp	Gene Function & Reference
AT1G17840	ABCG11	ATP-binding cassette G11	2	3	Cutin transport [88], vascular development [89].
AT1G67560	LOX6	Lipoxygenase 6	2	3	JA synthesis, wounding response [90], stress resistance [91].
AT3G12500	PR3	Pathogenesis-related protein 3, encodes basic chitinase	2	3	JA-mediated pathogen defence [59], [60].
AT3G45140	LOX2	Lipoxygenase 2	2	3	wounding response [92], JA synthesis and senescence [93].
AT4G15440	HPL1	Hydroperoxide Lyase 1	2	3	JA signaling stress response [94], [95].
AT3G14840	LIK1	LYSM RLK1-interacting kinase 1	6	3	JA-dependent pathogen defense [96].
AT4G20140	GSO1	GASSHO1, receptor-like kinase	6	3	Embryonic cuticle formation [97], [98].
AT5G46050	PTR3	Wound-induced peptide transporter	6	3	JA-mediated wounding response, pathogen defence [99], salt stress [100].
AT1G27730	ZAT10	Cys2/His2-type zinc finger protein	2,6	-	Drought stress, osmotic stress, heat stress, salt stress [101], [102].
AT3G25250	AGC2-1	AGC2 kinase 1	2,6	-	Oxidative stress [103], pathogen defence [104].
AT1G19640	JMT	Jasmonic acid carboxyl methyltransferase	2,6	3	Drought stress [64], cold stress [65].
Reproduction GO:0000003					
Gene ID	Gene Symbol	Gene Description	Cluster in Kd	Cluster in Kp	Gene Function & Reference
AT2G44190	EDE1	Microtubule-associated protein, endosperm defective 1	4	8	Microtubule function during seed development [105].
AT4G13560	UNE15	Late embryogenesis abundant proteins, Unfertilised embryo sac 15	4	8	Stress response [106].
AT5G10510	AIL6	Aintegumenta-like 6	4	8	Auxin-mediated flower development [107].
AT5G66730	IDD1	Indeterminate domain 1	8	8	Promote germination [87]

AT1G60420	NRX1	Nucleoredoxin 1	4,8	-	Pollen tube growth [108].
AT1G64625	LHL3	Lonesome highway like 3	4,8	-	Meiotic synchrony during reproduction [109], root development [110].
AT4G24580	REN1	ROP1 enhancer	4,8	-	Pollen tube development [111], [112].
AT4G37750	ANT	Aintegumenta	4,8	-	Plant defence [107], [113], Auxin-mediated flower development [114].
AT5G66460	MAN7	Endo-beta-mannase 7	4,8	-	seed germination [115], silique dehiscence [116].
AT4G24660	HB22	Homeobox protein 22	4,8	8	Embryo development [117], seed tolerance [118].
Regulation of hormone levels GO:0010817					
Gene ID	Gene Symbol	Gene Description	Cluster in Kd	Cluster in Kp	Gene Function & Reference
AT1G73590	PIN1	Auxin efflux carrier, PIN-formed 1	5	12	Establish embryo axis [119], Shoot and root development [120].
AT4G09160	PATL5	Patellin protein 5	5	12	Embryo patterning organogenesis, Stress response [121],
AT5G55540	TRN1	Tornado 1	5	12	Leaf patterning [122], root epidermal patterning [123], auxin transport [124].
AT5G65640	bHLH093	Beta HLH protein 93	5	12	Gibberellin-mediated reproductive growth [125].
AT1G19790	SRS7	Shi-related sequence 7	6	12	Flower development [126], [127].
AT1G68320	MYB62	MYB domain protein 62	6	12	Phosphate starvation response, gibberellin biosynthesis [128], [129].
AT1G75520	SRS5	Shi-related sequence 5	6	12	promotes photomorphogenesis [130], flower development [127], [131], lateral root formation [132].
AT2G26710	BAS1	PhyB activation tagged suppressor	6	12	Brassinosteroids metabolism [133], [134].
AT3G51060	STY1	Stylish 1	6	12	Auxin biosynthesis [135], flower development [131], [136].
AT2G34650	PID	Pinoid	5,6	-	Positive regulator of cellular auxin efflux [137], a negative regulator of auxin signaling [138].
AT2G47260	WRKY23	WRKY DNA-binding protein	5,6	-	Mediates PIN polarity [139], embryo development [140].
AT4G33090	APM1	Aminopeptidase M1	5,6	-	Auxin polar transport [141].
Response to karrikin GO:0080167					
Gene ID	Gene Symbol	Gene Description	Cluster in Kd	Cluster in Kp	Gene Function & Reference
AT1G06520	GPAT1	Glycerol-3-phosphate sn-2-acyltransferase	1	9	Pollen development [142], seed development [143], [144].
AT3G11600	GIR2	Plant-specific adapter protein	1	9	Root hair development [145], promote histone deacetylation [146].
AT3G47600	MYB94	Putative transcription factor	1	9	Cuticle formation [147], [148].

Supplementary Figure S1A

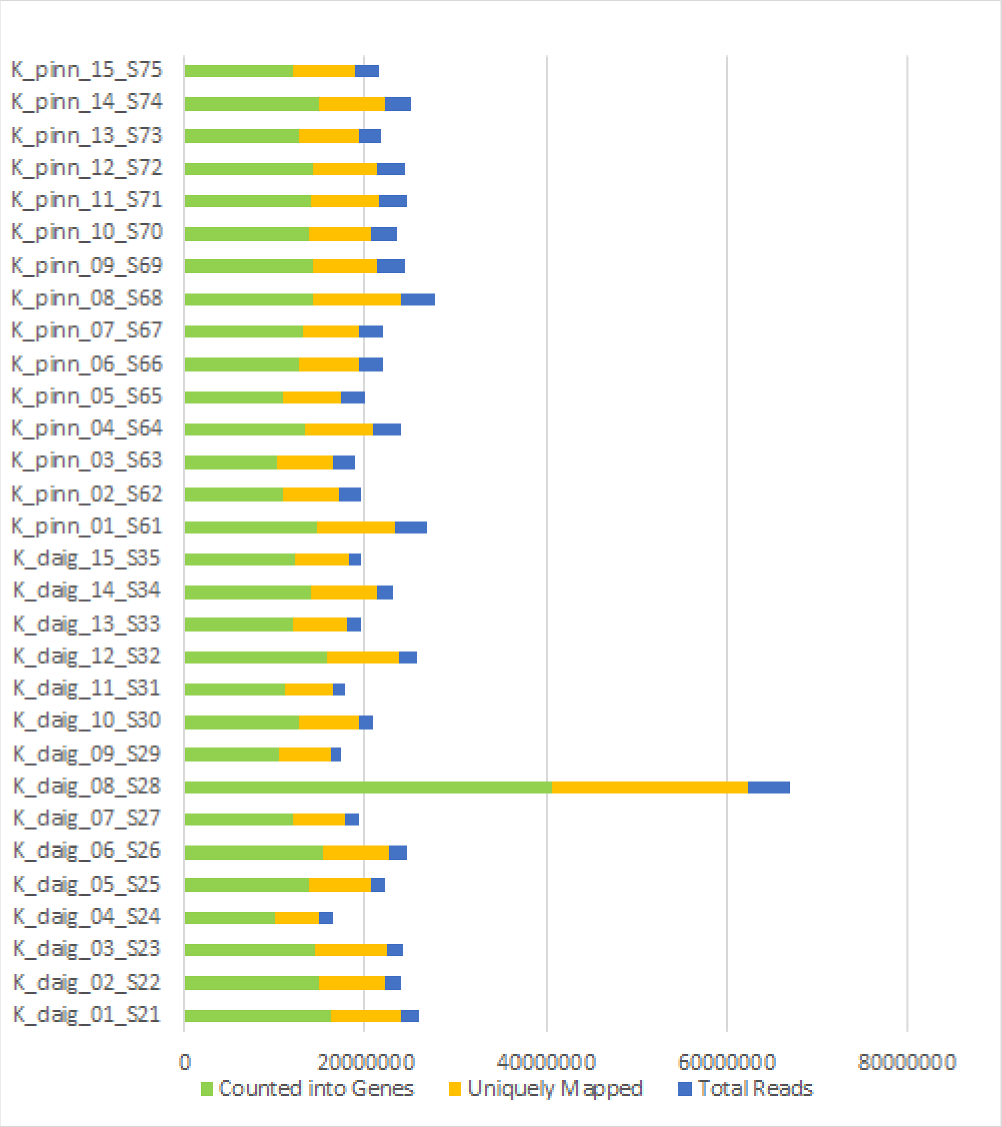

Supplementary Figure S1B

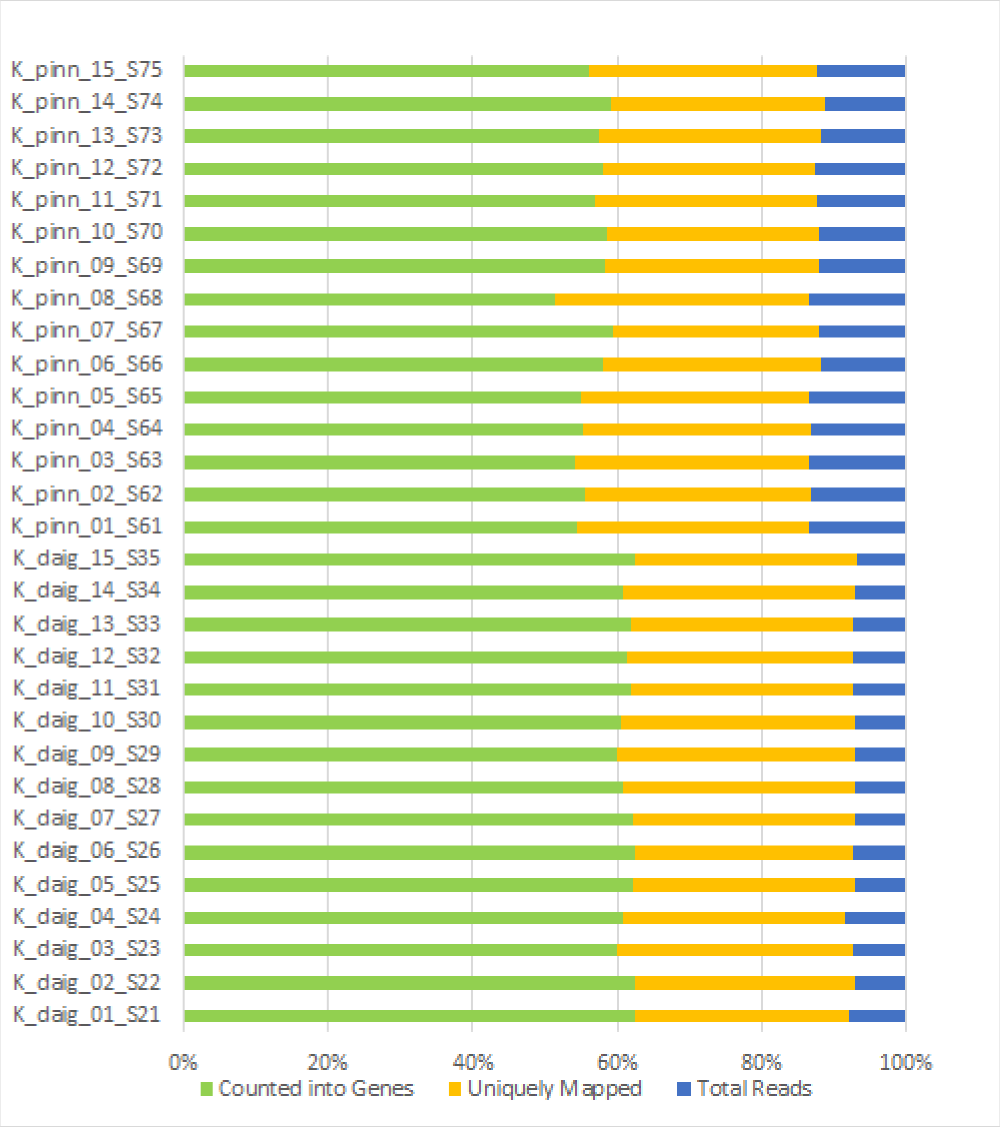

Supplement: Supplementary file 1 [file plants-11-01643-s001.zip › plants-1774495-supplementary.pdf]
